# Diagnosing possible infantile cow’s milk protein allergy in rural Africa, when history and physical examination are the only tools: a case report

**DOI:** 10.4076/1757-1626-2-6287

**Published:** 2009-07-17

**Authors:** Carsten Krüger, Isaack Malleyeck

**Affiliations:** 1 Department of Paediatrics, St.-Franziskus-HospitalRobert-Koch-Str. 55, D-59227 AhlenGermany; 2 Haydom Lutheran HospitalP.O. Mbulu, Haydom via MbuluTanzania

## Abstract

**Introduction:**

Cow’s milk protein allergy is common in infants from industrialised countries, but is rarely considered in developing countries due to its variable clinical presentation.

**Case presentation:**

We report on a Tanzanian male infant, who developed blood-stained stool when feeding fresh cow’s milk at the age of three months. After an initial diagnosis of amoebiasis, possible cow’s milk protein allergy was suspected. Further diagnostic work-up was not possible due to lack of resources. After elimination of cow’s milk from the diet, the infant recovered soon.

**Conclusion:**

Cow’s milk protein allergy should be considered more frequently in infants from developing countries, especially when they belong to agropastoralist tribes and are fed cow’s milk early.

## Introduction

In industrialised countries, blood-stained stool in infants is, besides other signs and symptoms (Table 1), quite a common symptom of cow’s milk protein allergy (CMPA), more so in bottle- than in exclusively breast-fed infants [1-3]. The prevalence of CMPA during the first twelve months of life is estimated to be around 5-15%, including all manifestations of CMPA, but figures in the lower range are probably more realistic [3]. Prevalence data from developing countries are not available. The few reports on CMPA in these countries are case reports from university hospitals in Asia or Northern Africa, [4,5] which have the same diagnostic tools as in industrialised countries and do not provide any prevalence figures because of selection bias.

**Table 1.** Common symptoms of cow’s milk protein allergy (CMPA) in infants (modified after Vandenplas et al. [3])

Organ system	Symptoms in severe CMPA	Symptoms in mild to moderate CMPA
Gastrointestinal	Failure to thrive due to chronic diarrhoea	Frequent regurgitation
	Refusal to feed	Vomiting
	Vomiting	Diarrhoea
	Iron deficiency anaemia due to occult/macroscopic blood loss	Constipation (with/without perianal rash)
		Blood in stool
	Endoscopically/histologically confirmed enteropathy or severe colitis	Iron deficiency anaemia
Respiratory (without signs of infection)	Acute laryngoedema	Runny nose, otitis media
	Bronchial obstruction with difficulty in breathing	Chronic cough
		Wheezing
Skin	Exudative or severe atopic dermatitis	Atopic dermatitis
	- with hypoalbuminaemia	Swelling of lips or eye lids (angio-oedema)
	- with failure to thrive	Urticaria unrelated to infection, drugs or other causes
	- with iron deficiency anaemia	
General	Anaphylaxis	Persistent colic or distress at least 3 days per week for more than 3 weeks

In resource-poor settings like rural Africa, infantile CMPA is hardly ever diagnosed as such although it might be as common as in other settings [1]. For example, blood-stained stool in infants and children (haematochezia, melaena, or dysentery in case of accompanying diarrhoea) is most frequently caused by parasitic, bacterial or viral infections [1,2]. Causative organisms are *Entamoeba histolytica*, *Shigella*, *Salmonella*, rotavirus and others, to mention just a few [1,2]. Other differential diagnoses are often not considered, but range from intussusception, Meckel’s diverticulum, Hirschsprung’s enterocolitis, polyps, haemorrhagic disease of the newborn, constipation, anal fissures (these two being the two most common), to allergic proctocolitis and food allergies like CMPA [2]. Besides not being considered in the differential diagnosis, the scarce or absent diagnostic tools contribute to the problem. Here we report on an infant with a possible diagnosis of CMPA from Tanzania.

## Case presentation

After an uneventful pregnancy, the male infant was delivered at term with a birth weight of 3.6 kg. He was the fourth child of healthy parents, the other three siblings being healthy. Allergies and atopic diseases were not reported. The family belonged to the Iraqw tribe whose members are mostly agropastoralists with ready access to cow’s milk [6-8]. This is frequently consumed by breastfeeding mothers and even given to young infants right after birth or during the following months, often in addition to breastfeeding [8]. Exact prevalence data on this custom are not available. The Iraqw live in a rural area in Northern Tanzania, and people rely on subsistence farming [9]. Medical care is sought either at local dispensaries or at a large, well-functioning church-owned hospital [9].

After delivery, the infant was exclusively breastfed for three months. The mother had him checked regularly in the mother-child-health clinic at the hospital. Vaccinations were administered according to the national plan. Because his mother had to resume work after this period of three months, in addition to breastfeeding she had him fed fresh cow’s milk. Shortly afterwards at the age of four months (weight 6.0 kg), the infant developed fresh blood-stained stools, sometimes with loose stools, but no diarrhoea. Fever or other symptoms were not present. At the mother-child-health clinic, *Entamoeba histolytica* was seen microscopically in a stool sample, thus a diagnosis of amoebiasis was made by the attending non-physician health worker. According to the national treatment plan, the infant was given a full course of metronidazole. Despite some improvement, again fresh blood-stained stool was repeatedly noted during the next weeks, and one month later *Entamoeba histolytica* was still found microscopically. In addition to a new course of metronidazole, cotrimoxazole was given then because of the suspicion of *shigellosis* (no microbiology facilities available at the hospital at that time). Another month later, the child presented with the same symptoms, again *Entamoeba histolytica* was seen in a stool sample, and now tinidazole, cotrimoxazole and chloroquine were prescribed by the attending non-physician health worker.

Because of his recurrent problem, at that time he was referred to the consultant paediatrician at the hospital. Here the history revealed for the first time that he had been given fresh cow’s milk throughout the last three months. It was suspected that another diagnosis could be responsible for this recurrent haematochezia. The general condition was fine, there was no diarrhoea, anaemia or fever, the weight was 7.5 kg. The clinical examination did not show any abdominal or perianal problems. Abdominal ultrasound was normal. As no laboratory facilities for further blood and stool analyses, total immunoglobulin E or specific antibodies were available apart from stool microscopy, and a skin prick test was not possible, a preliminary diagnosis of CMPA was made. Other signs and symptoms of CMPA (Table 1) were not present. The antibiotic and antimalarial treatment was stopped, and a dietary trial was initiated. Cow’s milk was excluded from the diet, the mother resumed exclusive breastfeeding with gradual introduction of cow’s milk free supplementary food, and after about one month the symptom of blood-stained stool had disappeared completely. The child thrived well and weighed 10.5 kg at the age of one year. During the second year of life, cow’s milk was introduced gradually again into the diet, but symptoms never re-occurred.

## Discussion

Admittedly, our diagnosis of possible infantile CMPA rests completely on history, physical examination and the success of the dietary elimination trial. Obviously, a laboratory workup with immunoglobulin E and skin prick tests could have supported our diagnosis more firmly, [2,3,10] but this was not possible due to technical and logistic (no such facilities in the whole of Tanzania available at that time) and financial constraints (the family was too poor for these rather expensive investigations). An endoscopic examination of the rectosigmoid was also not possible due to the lack of an appropriately small rectoscope. Nevertheless, the original diagnosis of amoebiasis was not very likely as the child did not have any other symptoms (fever, diarrhoea, vomiting etc.) and thrived well. In addition, retrospectively it could not be confirmed whether the laboratory assistant saw *Entamoeba histolytica* cysts or trophozoites, only the latter form being pathogenic [1]. Other infectious agents were not possible to exclude, but also not likely due to the good general condition and the missing clinical symptoms of recurrent gastrointestinal infection. Other abnormalities were not found.

Our diagnosis of CMPA is not definitely confirmed, as only 18% of infants with the symptom of blood-stained stool had CMPA in a recent study [11]. Still, we would like to stress that especially in tribes which feed cow’s milk to young infants, either for nutritional or cultural reasons, and of which are many in sub-Saharan Africa and elsewhere, this differential diagnosis should be considered [8,9,12,13]. But even in exclusively breastfed infants, CMPA is possible [2,3,14]. For neither group any prevalence figures are available for developing countries. A high index of suspicion is required, and in the absence of sophisticated laboratory facilities only a therapeutic dietary trial (and perhaps a skin prick test) will give the answer whether a correct diagnosis was made or not. If successful, cow’s milk and its products should only be re-introduced into the child’s diet after the age of 9-12 months, preferably after consultation with a physician. A supervised food challenge after a few weeks of elimination diet for confirmation of diagnosis, as suggested recently, [11,15] will often not be practical, given the difficult economic and structural circumstances in these countries. This is most likely only feasible in infants with severe manifestations of CMPA (Table 1).

## Conclusion

If CMPA is strongly suspected from history, socio-cultural circumstances, and clinical findings in a developing country setting with restricted or absent laboratory facilities, it is recommended to conduct a therapeutic trial with the exclusion of cow’s milk from the infant’s or mother’s (in case the infant is exclusively breastfed) diet.

## Abbreviation

CMPA: cow’s milk protein allergy
